# The Fungicidal Terpenoids and Essential Oil from *Litsea cubeba* in Tibet

**DOI:** 10.3390/molecules15107075

**Published:** 2010-10-13

**Authors:** Yu Yang, Jiazheng Jiang, Luobu Qimei, Xiaojing Yan, Junxia Zhao, Huizhu Yuan, Zhaohai Qin, Mingan Wang

**Affiliations:** 1 Department of Applied Chemistry, China Agricultural University, Beijing 100193, China; 2 Department of Chemistry, College of Sciences, Tibet University, Lhasa 850000, China; 3 Institute of Plant Protection, China Academy of Agricultural Sciences, Beijing 100193, China

**Keywords:** *Litsea cubeba*, Lauraceae, (6R)-3,7-dimethyl-7-hydroxy-2-octen-6-olide, litseacubebic acid, essential oil, fungicidal activity

## Abstract

A new C_9_ monoterpenoid acid (litseacubebic acid, **1**) and a known monoterpene lactone (6R)-3,7-dimethyl-7-hydroxy-2-octen-6-olide (**2**), along with three known compounds – vanillic acid (**3**), *trans*-3,4,5-trimethoxylcinnamyl alcohol (**4**), and oxonantenine (**5**) – were isolated with bioassay-guided purification from the fruit extract of *Litsea cubeba* collected in Tibet. The structure of **1** was elucidated by MS, ^1^H-NMR, ^13^C-NMR, COSY, HSQC, HMBC, NOE spectral data as 2,6-dimethyl-6-hydroxy-2*E*,4*E*-hepta-2,4-diene acid. Additionally 33 compounds were identified from the essential oil of *L. cubeba*. The preliminary bioassay results showed that **1** and **2** have good fungicidal activities against *Sclerotinia sclerotiorum, Thanatephorus cucumeris, Pseudocer-cospora musae* and *Colletotrichum gloeosporioides* at the concentration of 588 and 272 μM, and the essential oil has good fungicidal activities against *T. cucumeris* and *S. sclerotiorum*, with IC_50_ values of 115.58 and 151.25 μg/mL, repectively.

## 1. Introduction

*Litsea cubeba* (Lour.)Pers. is a plant of Lauraceae family. The essential oil from the fruit contains 75% citral, but the essential oil from the leaves contains more 1,8-cineole than citral [1]. In fact the compositions were different due to their location and collection times [2]. The essential oil is widely used as a flavor enhancer in foods, cosmetics and cigarettes; as raw material for the manufacture of citral, vitamins A, E and K, ionone, methylionone, and perfumes; and as an antimicrobial and insecticidal agent [3], and the complex and molecular microcapsules of *L. cubeba* oil with β-cyclodextrin and derivatives were reported recently [4]. The oral and dermal LD_50_ of the oils in mice were reported near 4,000 and 5,000 mg/kg of body weight, respectively. In addition lots of aporphine alkaloids [5,6,7,8,9], lactone [10], and flavone derivatives were isolated from the root, stem, barks of *L. cubeba*, and their structure-activity relationships against *AchE* for the aporphine alkaloids and their derivatives have been discussed in detail [11]. The crude extract of *L. cubeba* was screened in our laboratory and showed fungicidal activities. However, the ingredients of the essential oil, and the extracts from the fruit of *L. cubeba* produced in Tibet, and their fungicidal activities were unknown. In order to seek new lead fungicidal compounds, the components of essential oil, and extracts from the fruit of *L. cubeba* collected in Tibet, and their fungicidal activities are reported in this paper.

## 2. Results and Discussion

The light-yellow essential oil was obtained in 2.73% yield. The components were analyzed and identified with GC-MS and relative index (*RI* value) in the same column compared with standard mixtures of alkanes. The results showed that 33 compounds were present in this oil, namely limonol (44.2%), β-linalool (8.8%), 1,8-cineole (5.4%), elemicin (3.9%), methyleugenol (3.8%), esteragenol (2.8%), deoxygeraniol (2.6%), citronellal (2.5%), α-citral (2.1%), α-pinene (1.9%), myristicin (1.8%), β-Geraniol (1.6%), α-terpineol (1.4%), α-ocimene (1.3%), β-pinene (1.3%), terpinen-4-ol (0.8%), β-caryophyllene (0.7%), limonol acetate (0.7%), β-ocimene (0.6%), shikomol (0.6%), and the other thirteen minor compounds were 2-carene, 3-carene, *m*-cymene, *p*-cymene, γ-terpinene, 6,7-epoxy-linalool, rosenoxide, limonol formate, β-elemene, β-patchoulene, β-selinene, methylisoeugenol, β-caryophyllene oxide. The differences are limonol, β-linalool, 1,8-cineole, elemicin, and not citral, limonene, citronellal for the main components [1,2].

Bioassay-guided fractionation, macroporous resin column chromatography, silica gel chromatograph, and preparative TLC isolation of the EtOH extracts of the fruit of *L. cubeba* yielded five compounds. Four of them were characterized as (6*R*)-3,7-dimethyl-7-hydroxy-2-octen-6-olide (**2**) [12,13], vanillic acid (**3**) [14], *trans*-3,4,5-trimethoxyl-cinnamyl alcohol (**4**) [15], and aporphine alkaloid oxonantenine (**5**) [16] by m.p., [α]_D_, MS, ^1^H-NMR and ^13^C-NMR spectral data.

Litseacubebic acid (**1**) gave a molecular weight of 170 by ESI-MS (+) m/z: 193[M+Na]^+^, 209[M+K]^+^, and ESI-MS(-)m/z: 169[M-H]^-^. The molecular formula C_9_H_14_O_3_ was obtained by HR-FAB-MS m/z: 193.0846 [M+Na]^+^, C_9_H_14_O_3_Na, calcd. 193.0841. The ^1^H-NMR and ^13^C-NMR data indicated that one methyl (δ_H_, 1.96 d, *J* = 1.0 Hz, 3H; δ_C_: 12.50), one isopropanol methyl (δ_H_: 1.38 s, 6H; δ_C_: 29.69, 71.13), two double bond olefins (δ_H_: 7.27 dd, *J* = 11.0, 1.0 Hz, 1H; 6.58 dd, *J* = 11.0, 15.5 Hz, 1H; 6.20 d, *J* = 15.5 Hz, 1H; δ_C_: 121.97, 126.59, 139.45, 149.27), and one carboxylic acid carbonyl (δ_C_: 172.18) were present in the molecule of **1**. The correlations between the olefin protons at δ 7.27 and 6.58, 6.58 and 6.20 in the COSY spectrum of **1**, and the coupling constants of 11.0 and 15.5 Hz, showed they are the adjacent protons connected on the conjugated olefin. The long range allylic coupling of 1.0 Hz between the olefin protons at δ 7.27 and the methyl group at δ 1.96 was confirmed due to the correlation between them in the COSY spectrum of **1**. The carbon signals were assigned on the basis of HSQC and DEPT spectra of **1**. The positions of substituent groups were determined depending on the correlations between the proton at δ 1.96 and the carbon at δ 172.18, 126.59, and 139.45, the proton at δ 7.27 and the carbon at δ 149.27 and 12.50, the proton at δ 6.58 and the carbon at δ 71.13, the proton at δ 6.20 and the carbon at δ 71.13 and 29.69, the proton at δ 1.38 and the carbon at δ 149.27. The *E*-configuration of double bonds at C_2_ and C_4_ positions were determined due to the NOE enhancement of the proton at δ 6.58 when irridiating the proton at δ 1.96 in the NOE difference spectrum, and the 15.5 Hz coupling constant of the protons at C_4_ and C_5_. Thus the structure of **1** was elucidated to be 2,6-dimethyl-6-hydroxy-2*E*,4*E*-hepta-2,4-diene acid. To the best of our knowledge, it is found for the first time in Nature. As we know, 2,6-dimethyl-6-hydroxy-2*E*,4*E*- hepta-2,4-dienal (**6**) was found to be present in Labdanum oil [17], and the tradional Chinese medicine *Alpinia oxyphilla* Miq. [18]. Both **1** and **6** are C_9_ monoterpenes, which including the other C_9_ monoterpenoids such as dimetol originate from biosynthesis pathway of terpenoids, and **2** are relating structurally with citral and geranic acid existing in the essential oil of *L. cubeba*.


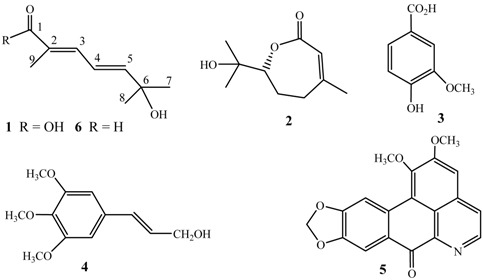


The fungicidal activities of terpenoids compounds **1,2,** and the essential oil (EO) were assayed against phytopathogens such as *Botrftis cinerea*, *Alternaria mali*, *A. alternate*, *Fusarium oxysporurn, F. moniliforme*, *F. graminearum*, *Phytophthora infestans*, *Sclerotinia sclerotiorum*, *Colletotrichum lindemuthianum*, *Verticillium dahliae*, *Thanatephorus cucumeris*, *Botryosphearia berengeriana*, *Pseudocer-cospora musae*, and *Colletotrichum gloeosporioides* at the concentrations of 588 μM (**1**), 272 μM (**2**), and for the essential oil 300 μg/mL. The data in Table 1 indicate that compound **1** showed 56.7%~86.7% inhibitory ratio against seven fungi, compound **2** showed 53.5%~81.8% inhibitory ratio against 13 fungi, while the essential oil showed excellent fungicidal activities against *S. sclerotiorum*, *T. cucumeris* and *A. mali* with 100%, 100%, and 83.2% inhibitory rate, respectively. However, the data in Table 1 also indicated that **1**, **2**, and the essential oil showed no significant inhibition against the other fungi. These results suggested that the activities of **2** were relatively broad spectrum, which indicated the lactone ring in **2** is a very important moiety for the fungicidal activities of this kind of terpenoid compounds. The further precise determination of the toxicities for the essential oil indicated that the IC_50_ values against *S. sclerotiorum* and *T. cucumeris* were 151.25 and 115.58 μg/mL, repectively.

**Table 1:** Inhibitory ratio (%) of **1** (588 μM), **2** (272 μM), **7**(52 μM)and EO (300 μg/mL) against plant fungi.

Fungi	EO	1	2	7	Fungi	EO	1	2	7
*B. cinerea*	49.8	-	72.0	100	*S. sclerotiorum*	100	86.7	81.8	100
*A. mali*	83.2	-	66.0	100	*C. lindemuthianum*	-	-	62.3	-
*A. alternate*	-	57.8	70.9	-	*V. dahliae*	-	-	66.6	-
*F. oxysporurn*	-	56.7	53.5	100	*T. cucumeris*	100	76.8	80.2	100
*F. moniliforme*	-	-	55.6	100	*B. berengeriana*	-	59.2	-	-
*F. graminearum*	-	-	73.8	100	*P. musae*	-	78.9	65.5	-
*P. infestans*	-	-	66.4	100	*C. gloeosporioides*	-	71.2	71.1	100

## 3. Experimental

### 3.1. General

Melting points were measured on a Yanagimoto apparatus and are uncorrected. Optical rotations were measured on a Perkin-Elmer 341 polarimeter. ^1^H-NMR, ^13^C-NMR, COSY, HMQC, HMBC, and NOESY spectra were recorded on Brüker DRX 500 and DPX 300 NMR Spectrometers with CDCl_3_ as solvent and TMS as internal standard. ESI-MS data were analyzed with an Agilent LCQ LC-MSD ion-trap mass spectrometer. HR-FAB-MS were obtained on Brüker Apex II mass spectrometer using nitrobenzoyl alcohol and sodium chloride as matrix. The solvents were analytical grade and without treatment before usage. Agilent 6890GC 5973I MSD GC-MS spectrometer with HP-5MS column (0.25 mm × 30 m × 0.25 μm) was used. The standard mixture of alkanes was provided friendly by Dr. Zhou Ligang, China Agricultural University. The NIST Library (2002 Ed.) for mass spectra search and *RI* values were used for peak identification of the essential oil analysis.

### 3.2. Plant Material

The fruit of *Litsea cubeba* was collected in Yigong workshop, Bomi County, Tibet, China on Oct., 2008, and authenticated by Prof. Wang Li of the Department of Plant Sciences, College of Biology, China Agricultural University. The voucher specimens were deposited at the Department of Plant Sciences, China Agricultural University.

### 3.3. Extraction and Isolation

The 10.93 g essential oil was obtained from 400 g of *L. cubeba* fruit by steam distillation for 12 h and dried over anhydrous MgSO_4_. The dried and pulverized fruit (4 Kg) of *L. cubeba* was extracted three times with 95% C_2_H_5_OH one week each time. The extracted material was dispersed into water, and re-extracted three times with petroleum ether and EtOAc, respectively. The EtOAc extract was evaporated to afford a residue (17 g). The water phase was absorbed on a macroporous resin column, washed glucose with water, and eluted with 95% C_2_H_5_OH, evaporated the eluents under reduced pressure to give a residue (10 g).

This EtOAc extract (15 g) was chromatographed on a silica gel (560 g, 200–300 mesh) column using EtOAc-petroleum ether as eluents to give eight fractions. Active fraction 5 (580 mg) was repeatedly prepared by preparative thin layer chromatograph, which was developed with petroleum ether-diethyl ether-acetic acid (*V_1_*:*V_2_*:*V_3_* = 90:30:2) two times to afford compounds **1** (20 mg) and **3** (16 mg). Active fraction 6 (600 mg) was repeatedly prepared by preparative thin layer chromatograph, which was developed with petroleum ether-diethyl ether-acetic acid (*V_1_*:*V_2_*:*V_3_* = 120:30:2) two times to afford fractions 6-1 and 6-2. Fraction 6-2 was further prepared by preparative thin layer chromatograph, which was developed with petroleum ether-acetone (*V_1_*:*V_2_*: = 80:20) two times to give compound **4** (15 mg). Active fraction 7 (450 mg) was repeatedly prepared by preparative thin layer chromatograph, which was developed with petroleum ether-diethyl ether-acetic acid (*V_1_*:*V_2_*:*V_3_* = 50:75:2) two times to afford fractions 7-1, 7-2, 7-3 and 7-4. Fractions 7-3 and 7-4 were combined after check with TLC, further prepared by preparative thin layer chromatograph, which was developed with hexane-chloroform-methanol (*V_1_*:*V_2_*:*V_3_* = 30:50:10) three times to give compound **2** (15 mg). 

The water phase extract (10 g) plus water-insoluble part (50 g) was chromatographed on a silica gel column (1,000 g, 200–300 mesh) using EtOAc- methanol as eluents to give 31 fractions. Fraction 3 (5 g) was re-chromatographed on a silica gel (200–300 mesh) column using EtOAc-petroleum ether (1:9-9:1) to give 3-1~3-8 fractions. Fraction 3–7 (300 mg) was prepared by preparative thin layer chromatograph, which was developed with petroleum ether-diethyl ether-acetic acid (*V_1_*:*V_2_*:*V_3_* = 60:30:2) two times to afford fractions 3-7-1, 3-7-2, and 3-7-3. Fraction 3-7-2 (70 mg) was further prepared by preparative thin layer chromatograph, which was developed with hexane-chloroform-methanol (*V_1_*:*V_2_*:*V_3_* = 50:50:25) two times to afford compound **1** (7 mg). Combined fractions 8~19 (4.8 g) was re-chromatographed on a silica gel (200-300 mesh) column using EtOAc-methanol to give 8-1~8-7 fractions. Fraction 8-7-6 (180 mg) was further prepared by preparative thin layer chromatograph, which was developed with hexane-chloroform-methanol (*V_1_*:*V_2_*:*V_3_* = 40:60:20) two times to afford compound **5** (4.5 mg).

*Litseacubebic acid* (**1**): white solid, m.p.48~50 °C. ESI-MS (+) m/z: 193[M+Na]^+^, 209[M+K]^+^, ESI-MS(-)m/z: 169[M-H]^-^. HR-FAB-MS (+) m/z: 193.0846 [M+Na]^+^, C_9_H_14_O_3_Na, calcd. 193.0841. ^1^H- NMR (CDCl_3_, 500 MHz) δ: 7.27 (dd, *J* = 11.0, 1.0 Hz, 1H), 6.58 (dd, *J* = 11.0, 15.5 Hz, 1H), 6.20 (d, *J* = 15.5 Hz, 1H), 1.96 (d, *J* = 1.0 Hz, 3H), 1.38 (s, 6H); ^13^C-NMR (CDCl_3_, 125 MHz) δ: 172.18(C_1_), 126.59(C_2_), 139.45(C_3_), 121.97(C_4_), 149.27(C_5_), 71.13(C_6_), 29.69(C_7_/C_8_), 12.50(C_9_).

*(6R)-3,7-dimethyl-7-hydroxy-2-octen-6-olide* (**2**): light yellow liquid, [α]_D_^25^ = -58.8°. ESI-MS(+)m/z: 185[M+H]^+^, 207[M+Na]^+^. ^1^H-NMR (CDCl_3_, 300 MHz) δ: 5.86 (brq, *J* = 1.2 Hz, 1H), 4.04 (dd, *J* = 2.3, 8.9 Hz, 1H), 2.52 (dt, *J* = 17.9, 5.6 Hz, 2H), 2.33~2.38 (m, 1H), 2.10~2.13 (m, 1H), 1.93~1.95 (m, 1H), 1.97 (brs, 3H), 1.27 (s, 3H), 1.25 (s, 3H); ^13^C-NMR (CDCl_3_, 75 MHz) δ: 168.08, 154.68, 118.34, 84.38, 71.67, 33.51, 27.28, 26.10, 25.21, 24.62.

*Vanillic acid* (**3**): white solid, m.p.203~206 °C. ESI-MS(-)m/z: 167[M-H]^-^, 152[M-H-CH_3_]^-^, m/z137[M-OCH_3_]^-^. ^1^H-NMR (CDCl_3_, 300 MHz) δ: 7.72 (dd, *J* = 8.3, 1.9 Hz, 1H), 7.59 (d, *J* = 1.9 Hz, 1H), 6.97 (d, *J* = 8.3 Hz, 1H), 3.94 (s, 3H); ^13^C-NMR (CDCl_3_, 75 MHz) δ: 170.59, 150.79, 146.21, 125.18, 121.18, 114.20, 112.12, 56.14.

*trans*-*3,4,5-Trimethoxyl-cinnamyl alcohol* (**4**): light yellow solid, m.p.107~108 °C. ESI-MS(+)m/z: 209[M-CH_3_]^+^, 225[M+H]^+^, 247[M+Na]^+^. ^1^H-NMR (CDCl_3_, 300 MHz) δ: 6.61 (s, 2H), 6.53 (d, *J* = 15.9 Hz, 1H), 6.28 (dt, *J* = 5.7, 15.9 Hz, 1H), 4.32 (dd, *J* = 5.7, 1.4 Hz, 2H), 3.86(s, 3H), 3.84(s, 6H). ^13^C-NMR (CDCl_3_, 75 MHz) δ: 153.25, 138.00, 132.42, 131.00, 128.01, 103.54, 63.48, 60.86, 56.02.

*Oxonantenine* (**5**): red powder, m.p.224~225 °C. ESI-MS(+)m/z: 336[M+H]^+^, 358[M+Na]^+^. ^1^H NMR (CDCl_3_, 300 MHz) δ: ^1^H-NMR (CDCl_3_, 300 MHz) δ: 8.90 (d, *J* = 5.4 Hz, 1H), 8.68 (s, 1H), 8.00 (s, 1H), 7.78 (d, *J* = 5.4 Hz, 1H), 7.20 (s, 1H), 6.15 (s, 1H), 4.09(s, 3H), 4.01(s, 3H). ^13^C-NMR (CDCl_3_, 75 MHz) δ: 180.13, 156.86, 153.12, 151.64, 148.39, 145.29, 145.02, 139.27, 135.32, 131.02, 128.73, 123.39, 114.05, 107.85, 107.74, 106.21, 102.12, 60.69, 56.20.

### 3.4. Bioassay of Fungicidal Activities

Fungicidal activities of compounds **1**, **2** and the essential oil against *B. cinerea*, *A.mali*, *A. alternate*, *F. oxysporurn*, *F. moniliforme*, *F. graminearum*, *P. infestans*, *S. sclerotiorum*, *C. lindemuthianum*, *V. dahliae*, *T. cucumeris*, *B. berengeriana*, *P. musae*, and *C. gloeosporioides* were evaluated using the mycelium growth rate test [19]. The culture media with known concentration of the test compounds were obtained by mixing the solution of **1**, **2**, and the essential oil in acetone with potato dextrose agar (PDA), on which fungus cakes were placed. The blank test was made using acetone and carbendazim (**7**) was used as positive control. The culture was incubated at 25 ± 0.5 °C. Three replicates were performed. After the mycelium in the blank grew completely, the diameter of the mycelium was measured and the inhibitory ratio calculated according to the following formula. In which I is the inhibitory ratio, P_0_ is the average diameter of the mycelium in the blank, and P_1_ is the average diameter of the mycelium in the presence of the test samples. Mean measurements were calculated from the three replicates. The IC_50_ values were calculated using linear relation between the inhibitory probability and concentration logarithm according to the method outlined by Finney [20].


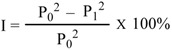


## 4. Conclusions

A new C_9_ monoterpenoid acid (litseacubebic acid, **1**), and a known monoterpene lactone (6*R*)-3,7-dimethyl-7-hydroxy-2-octen-6-olide were isolated from the fruit extract of *Litsea cubeba* collected in Tibet along with vanillic acid, *trans*-3,4,5-trimethoxylcinnamyl alcohol, and oxonantenine. The structure of **1** was elucidated as 2,6-dimethyl-6-hydroxy-2*E*,4*E*-hepta-2,4-diene acid. Thirty three compounds were also identified from the essential oil of *L. cubeba*. The preliminary bioassay results showed that **1** and **2** have good fungicidal activities against *S. sclerotiorum, T. cucumeris, P. musae* and *C. gloeosporioides* at the concentration of 588 and 272 μM, and the essential oil have good fungicidal activities against *T. cucumeris* and *S. sclerotiorum* with the IC_50_ 115.58 and 151.25 μg/mL, repectively.

## References

[1] Jiang Z., Akhtar Y., Bradbury R., Zhang X., Isman M.B. (2009). Comparative toxicity of essential oils of *Litsea pungens* and *Litsea cubeba* and blends of their major constituents against the cabbage Looper, *Trichoplusia ni*. J. Agric. Food Chem..

[2] Bighelli A., Muselli A., Casanova J. (2005). Chemical variability of *Litsea cubeba* leaf oil from Vietnam. J. Essent. Oil Res..

[3] Luo M., Jiang L.K., Zou G.L. (2005). Acute and genetic toxicity of essential oil extracted from *Litsea cubeba* (Lour.) Pers. J. Food Prot..

[4] Wang Y., Jiang Z.T., Li R. (2009). Complexation and molecular microcapsules of *Litsea cubeba* essential oil with β-cyclodextrin and its derivatives. Eur. Food Res. Technol..

[5] Tomita M., Lu S.T., Lan P.K., Lin F.M. (1965). Studies on the alkaloids of Formosan lauraceous plants. V. alkaloids of *Litsea cubeba* Pers. Yakugaku Zasshi.

[6] Wu Y.C., Liou J.Y., Duh C.Y., Lee S.S., Lu S.T. (1991). Studies on the alkaloids of Formosan Lauraceae plants. 32. Litebamine, a novel phenanthrene alkaloid from *Litsea cubeba*. Tetrahedron Lett..

[7] Lee S.S., Lin Y.J., Chen C.K., Liu K.C.S., Chen C.H. (1993). Quaternary alkaloids from *Litsea cubeba* and Cryptocarya konishii. J. Nat. Prod..

[8] Lee S.S., Chen C.K., Huang F.M., Chen C.H. (1996). Two dibenzopyrrocoline alkaloids from *Litsea cubeba*. J. Nat. Prod..

[9] Huang C.H., Huang W.J., Wang S.J., Wu P.H., Wu W.B. (2008). Litebamine, a phenanthrene alkaloid from the wood of *Litsea cubeba*, inhibits rat smooth muscle cell adhesion and migration on collagen. Eur. J. Pharmacol..

[10] Min B.S., Lee S.Y., Kim J.H., Kwon O.K., Park B.Y., An R.B., Lee J.K., Moon H.I., Kim T.J., Kim Y.H., Joung H., Lee H.K. (2003). Lactones from the leaves of *Litsea japonica* and Their anti-complement activity. J. Nat. Prod..

[11] Chiou C.M., Kang J.J., Lee S.S. (1998). Litebamine N-Homologues: Preparation and Anti-acetylcholine sterase Activity. J. Nat. Prod..

[12] Volcho K.P., Yarovaya O.I., Kurbakova S., Korchajina D.V., Barkhash V.A., Salakhutdinov N.F. (2007). Synthesis of epoxy dinitriles from citral and their acid-catalyzed transformations. Russ. J. Org. Chem..

[13] Gallardo G.L., Pena N.I., Cabrera G.M. (2008). Neric acid derivatives produced by the honey bee fungal entomopathogen *Ascosphaera apis*. Phytochemistry Lett..

[14] Miyazawa L.M., Oshima T., Koshio K., Itsuzaki Y., Anzai J. (2003). Tyrosinase inhibitor from black rice bran. J. Agric. Food. Chem..

[15] Matsuura H., Miyazaki H., Asakawa C., Amano M., Yoshihara T., Mizutani J. (2004). Isolation of β-glucosidase inhibitors from hyssop (Hyssopus officinalis). Phytochemistry.

[16] Chiu S.Y.C., Dobberstein R.H., Fong H.H.S., Farnsworth N.R. (1982). Oxoaporphine alkaloids from *Siparuna gilgiana*. J. Nat. Prod..

[17] Weyerstahl P., Marschall H., Weirauch M., Thefeld K., Surberg H. (1998). Constituents of commercial labdanum oil. Flavour Fragr. J..

[18] Xu J., Tan N., Zeng G., Han H., Huang H., Ji C., Zhu M., Zhang Y. (2009). Studies on chemical constituents in fruit of *Alpinia oxyphylla*. China J. Chin. Materia Medica.

[19] Chen N.C. (1991). The Bioassay Technologies for Pesticides.

[20] Finney S. (1978). Probit Analysis.

